# Homocysteine and Carotid Plaque Stability: A Cross-Sectional Study in Chinese Adults

**DOI:** 10.1371/journal.pone.0094935

**Published:** 2014-04-15

**Authors:** Xin Yang, Yong Zhou, Chao Liu, Xiang Gao, Anxin Wang, Yuming Guo, Wen Li, Xingquan Zhao, Wannian Liang

**Affiliations:** 1 Department of General Practice, School of General Practice and Continuing Education, Capital Medical University, Beijing, China; 2 Department of Neurology, Beijing Tiantan Hospital, Capital Medical University, Beijing, China; 3 Department of Neurosurgery, the Second Hospital of Jilin University, Changchun, China; 4 Channing Laboratory, Department of Medicine, Brigham and Women’s Hospital, and Harvard Medical School, Boston, Massachusetts, United States of America; 5 Department of Nutrition, Harvard University School of Public Health, Boston, Massachusetts, United States of America; 6 Department of Epidemiology and Biostatistics, University of Queensland School of Population Health, Brisbane, Queensland, Australia; 7 Department of Ultrasound, Kailuan Hospital, Hebei United University, Tangshan, China; 8 National Health and Family Planning Commission of People’s Republic of China, Beijing, China; University of Louisville, United States of America

## Abstract

**Background and Purpose:**

This study aimed to explore the possible association of plasma total homocysteine with carotid plaque stability.

**Methods:**

A cross-sectional study was conducted from 2010 to 2011. A stratified random sample of 2,919 Chinese participants aged 40 years or older was enrolled. Plasma total homocysteine levels were measured and carotid plaques were evaluated by ultrasonography. Logistic regression model was used to analyze the association of homocysteine levels to the progression of carotid plaque development, while adjusting for demographics and vascular risk factors.

**Results:**

The mean level of plasma homocysteine in the subjects was 14.9 µmol/l. Along with increase in homocysteine level, the risk of advanced carotid plaque elevated (odds ratio = 1.28; 95% confidence interval = 1.09–1.51) after adjusting for age, sex, and other potential confounders. Stratified by sex, higher homocysteine level was strongly associated with advanced carotid plaque in men (OR = 1.41; 95% confidence interval = 1.17–1.70), but not in women.

**Conclusion:**

The findings suggest that plasma level of homocysteine may be associated with advanced carotid plaque, which constitutes high risks of stroke, in male Chinese adults.

## Introduction

Stroke is the second leading cause of preventable mortalities and one of the leading causes of long-term disability [1]. In China, stroke and ischemic heart disease were the leading causes of DALYs (disability-adjusted life-year) in 2010 [2]. The medical management of ischemic stroke becomes a heavy burden to the modern health care system worldwide [3]. Ischemic stroke is usually caused by acute thrombosis, which is triggered by unstable atherosclerotic plaque instead of gradually progressive luminal narrowing [4], [5]. Previous studies already have found that homocysteine is significantly associated with ischemic stroke [6]–[12]. In 2013, the result of Northern Manhattan study (NOMAS) presented that elevated homocysteine is independently associated with plaque morphology and increased plaque area [13]. Accumulating studies mainly focus on relationships between homocysteine and vascular diseases, especially cardiovascular diseases in Caucasian population. So far, however, the relationship between homocysteine and vulnerable carotid plaque has not been investigated. Since unstable carotid plaque is a significant subclinical marker of stroke risk [14], [15], the main purpose of the present study, a cross-sectional study conducted in a community-based population in Northern China, is to analyze the relationship between the complexity of carotid plaque and the total plasma homocysteine level.

## Methods

### Study Design and Population

This study was a community-based cross-sectional study in Chinese adults derived from a reference population of the Kailuan Cohort Study. The subjects in the Kailuan cohort study were enrolled from a population of 101,510 employees (81,110 males and 20,400 females) of Kailuan (Group) Co. Ltd, a large coal mine industry in Hebei Province, China. Standard protocols were described previously [16], [17]. From June 2010 to June 2011, using stratified random sampling method by age and sex, 7,000 subjects aged ≥40 years were randomly selected. Among them, 5,852 signed informed consents and 5,816 individuals completed baseline data collection. Among these 5,816 participants, 401 subjects failed to meet the following inclusion criteria and therefore were excluded from this study: (1) No history of stroke, transient ischemic attack at baseline by a validated questionnaire, (2) Absence of neurologic deficits for stroke which was estimated by experienced doctors, (3) Fasting total plasma homocysteine has been examined. Among the 5,415 participants enrolled in the study, 2,919 were selected for carotid plaque assessed by ultrasonographic examination. The study was approved by the Ethics Committees of the Kailuan General Hospital and Beijing Tiantan Hospital. All participants signed informed consent.

### Assessment of Homocysteine

Blood samples were drawn by trained phlebotomists from the subjects after overnight fasting. The venous blood samples in tubes containing trisodium ethylenediaminetetraacetic acid were immediately placed on ice after antecubital venipuncture. Blood samples were then centrifuged for 10 minutes at 3000 rotations per minute at 25°C. After separation, plasma samples were used within 4 hours. Fasting plasma glucose was measured using the hexokinase/glucose-6-phosphate dehydrogenase method. Total cholesterol and triglyceride were measured enzymatically according to the manufacturer’s instruction (interassay coefficient of variation <10%; Mind Bioengineering Co. Ltd., Shanghai, China). All biochemical variables were measured using an autoanalyzer (Hitachi 747; Hitachi, Tokyo, Japan) at the central laboratory of the Kailuan General Hospital. Hyperhomocysteinemia was defined as plasma total homocysteine levels no less than 15 µmol/l [18].

### Assessment of Carotid Plaque

**Figure 1:**
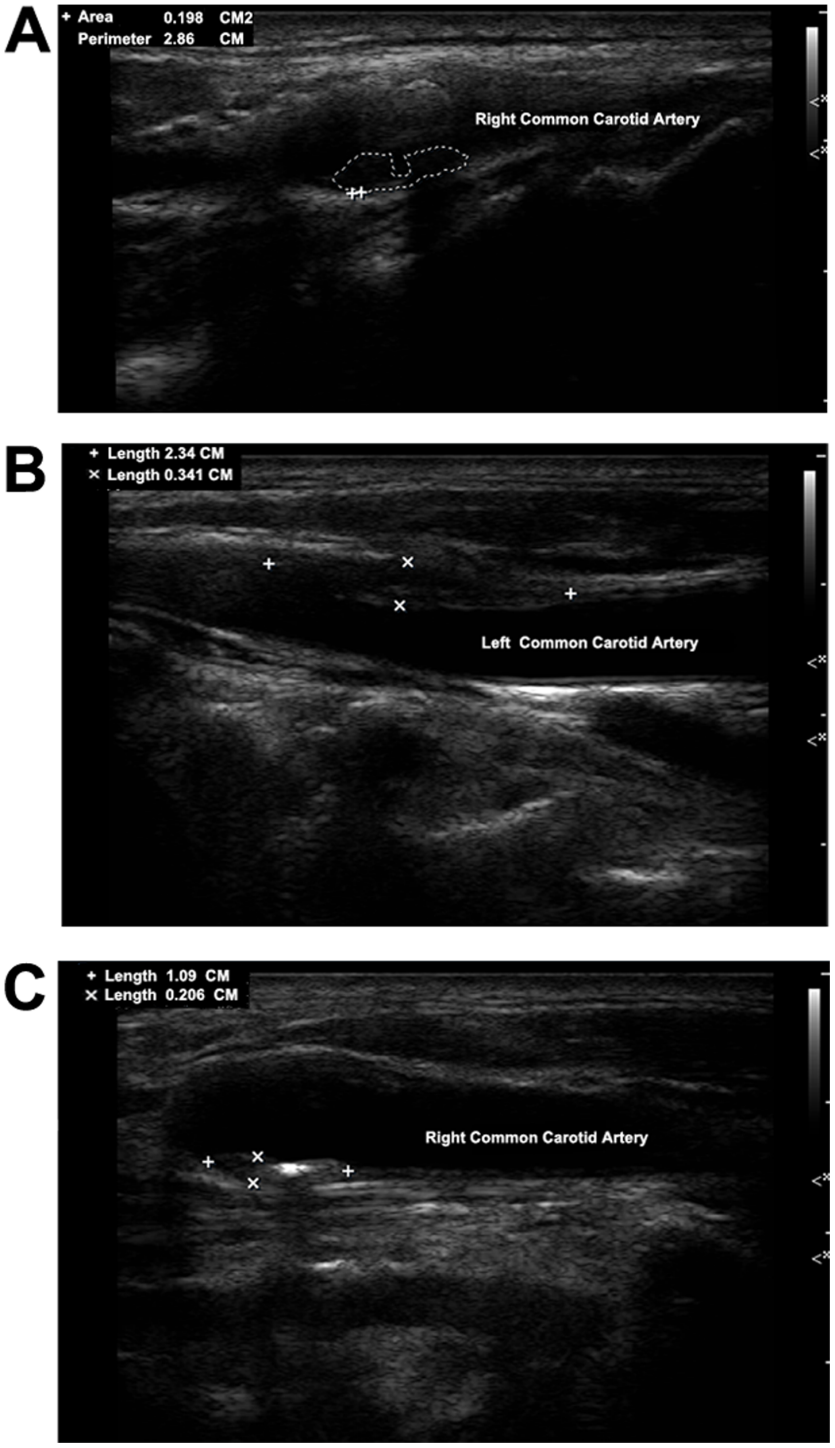
Representative ultrasound images for different types of advanced carotid plaques in study participants. A represents ulcerated plaque; B represents plaque with low-level echo; C represents plaque with heterogeneous echo.

Carotid plaques were evaluated to assess the complexity and advancement by trained and certified sonographers with ultrasounds (Philips iU-22 ultrasound system, Philips Medical Systems, Bothell, WA). Bilateral carotid arteries including common carotid arteries, carotid bifurcation, internal carotid artery, external carotid artery, were all examined with the patient in a supine position, head turning to the contralateral side. Both sides of carotid arteries were extensively evaluated. Carotid plaque was defined as a focal structure encroaching into the arterial lumen of at least 0.5 mm or 50% of the surrounding IMT value, or demonstrated as a thickness of 1.5 mm from the intima-lumen interface to the media adventitia interface. The carotid ultrasound examination results were then reviewed by two independent operators. Discrepancies between their evaluations were resolved by consensus. In this study, advanced or complicated carotid plaques were defined based on: (1) plaques with incomplete fibrous cap or ulcerated plaques, according to the plaque morphology, and (2) plaques with low-level or heterogeneous echoes, according to the plaque echodensity. Representative ultrasound images for different types of plaques were provided (Figure 1).

### Assessment of Demographic Variables and Cardiovascular Risk Factors

Information on demographic variables (e.g. age, sex and previous history of diseases) was collected via a questionnaire study. The participants were classified into two groups based on their ages: 40–59 years old group and those aged ≥60 years. The information of disease history mainly included hypertension, diabetes mellitus and hyperlipidemia. Questionnaires were also used to collect information on smoking history, which was classified as “yes”, or “no” according to self-reported information. Body weights (accurate to 0.1 kg) and heights (accurate to 0.1 cm) were measured, and the body mass indexes (BMI) were calculated. Hypertension was defined as presence of a history of hypertension, or using antihypertensive medication, or a SBP≥140 mmHg, or a DBP≥90 mmHg. Diabetes mellitus was defined as a self-reported history, currently treated with insulin or oral hypoglycemic agents, or fasting blood glucose level ≥7.0 mmol/L (126 mg/dl). Hyperlipidemia was defined as a self-reported history, current use of cholesterol lowering medicine, or total cholesterol level ≥5.7 mmol/L (220 mg/dl) or triglyceride ≥1.7 mmol/L (150 mg/dl). All the blood tests were done at the central laboratory of the Kailuan hospital.

### Statistical Analyses

Continuous variables were described by mean ± standard deviation (SD) and compared by ANOVA analysis. Categorical variables were described as percentages and compared using Chi Square tests. Logistic regression was used to evaluate the relationship between level of plasma total homocysteine and carotid plaque stability by calculating the crude odds ratio (OR) and adjusted OR, with 95% confidence interval (CI).

The well-known and possible risk factors of stroke such as age, sex, BMI, current smoking, alcohol use, hypertension, diabetes mellitus and hyperlipidemia, were adjusted in the statistical analysis. All statistical tests were 2-sided, and a significant level was set as *P*<0.05. SAS (version 9.1, SAS Institute, Cary, North Carolina, USA) software was used to perform data analyses.

## Results

### Characteristics and Risk Factors Associated with Carotid Plaque Stability

Table 1 shows the distribution of baseline cohort characteristics amongst those individuals with stable or advanced carotid plaque. Among the 2,919 participants, the mean concentration of homocysteine in elderly subjects (≥60 years old) was 19.3±9.8 µmol/l, while 15.6±9.7 µmol/l in the middle-aged subjects (40–59 years old). The level of plasma total homocysteine was higher in men than that in women (19.2±10.4 µmol/l versus 12.6±6.6 µmol/l respectively, P<0.01). By definition [18], 48.8% of the total subjects had hyperhomocysteinemia (41.3% in male and 7.5% in female). Advanced carotid plaque was detected in 1,517 (52%) participants. Among them, 76.1% were male. No significant association was observed between carotid plaque and BMI, alcohol use and hyperlipidemia.

**Table 1:** Basic characteristics of participants according to the carotid plaque stability.

	Overall participants characteristics (N = 2919)	Carotid Plaque Stability		P-value
		Stable (N = 1402)	Advanced (N = 1517)	
Mean age ± SD (years)	60.1±12.4	56.5±11.2	63.4±12.5	<0.01
Men,%	71.4	66.2	76.1	<0.01
Mean BMI ± SD (kg/m^2^)	24.9±3.2	24.9±3.2	24.9±3.3	0.85
Current Smoker, %	44.5	42.6	46.3	0.05
Heavy drinker, %	14.9	14.7	15.1	0.76
Hypertension, %	59.3	54.9	63.3	<0.01
Diabetes mellitus, %	16.3	14.8	17.7	0.03
Hyperlipidemia, %	54.2	52.6	55.7	0.09
Mean Homocysteine ± SD (µmol/l)	17.3±9.9	16.3±9.6	18.3±10.1	<0.01

SD, standard deviation; BMI, body mass index;

### Association between Total Homocysteine Levels and Carotid Plaque Stability

As shown in Table 2, an association of increased levels of total homocysteine with carotid plaque vulnerability in a mild dose-response manner was identified. In all 3 multivariable-adjusted models, higher levels of homocysteine were significantly associated with the advancement of carotid plaque. The crude ORs for advanced carotid plaque prevalence increased along with elevation of homocysteine level. After adjusting for age, sex, smoking, hypertension and diabetes mellitus, the association remained evident. However, in this study, hyperhomocysteinemia was not found to be significantly associated with the development of advanced carotid plaque in the female participants.

**Table 2:** ORs and 95%CIs for advanced carotid plaque according to homocysteine levels.

	Homocysteine		P-value	continuous
	<15 µmol/l	≥15 µmol/l		
Crude Model OR(95%CI)	Ref	1.62(1.40–1.87)	<0.01	1.02(1.01–1.03)
Model 1OR(95% CI)^1^	Ref	1.28(1.09–1.51)	<0.01	1.01(1.00–1.02)
Model 2OR(95% CI)^2^	Ref	1.28(1.09–1.51)	0.05	1.01(1.00–1.02)
**Sex**				
Female				
Model 1OR(95% CI)^3^	Ref	1.06(0.75–1.49)	0.73	1.01(0.98–1.03)
Model 2OR(95% CI)^4^	Ref	1.05(0.75–1.49)	0.77	1.00(0.98–1.03)
Male				
Model 1OR(95% CI)^3^	Ref	1.40(1.17–1.69)	<0.01	1.01(1.00–1.02)
Model 2OR(95% CI)^4^	Ref	1.41(1.17–1.70)	<0.01	1.01(1.00–1.02)
**Age**				
40–59 y				
Model 1OR(95% CI)^5^	Ref	1.37(1.10–1.72)	<0.01	1.01(1.00–1.02)
Model 2OR(95% CI)^6^	Ref	1.35(1.08–1.70)	<0.01	1.01(1.00–1.02)
≥60 y				
Model 1OR(95% CI)^5^	Ref	1.26(0.99–1.60)	0.06	1.01(0.99–1.02)
Model 2OR(95% CI)^6^	Ref	1.28(1.01–1.62)	0.04	1.01(0.99–1.02)

OR, odds ratio; CI, confidence interval;

1 Adjusted for age (year) and sex.

2 Adjusted for age (year), sex, hypertension, diabetes mellitus, current smoker.

3 Adjusted for age (year).

4 Adjusted for age (year), hypertension, diabetes mellitus, current smoker.

5 Adjusted for sex.

6 Adjusted for sex, hypertension, diabetes mellitus, current smoker.

## Discussion

To our knowledge, this is the first observational cohort study to explore the potential relationship between plasma homocysteine levels and development of carotid plaques in a Chinese adult population. Our data identified a significant association between hyperhomocysteinemia and the development of advanced carotid plaques in this population with an OR of 1.28 (95% CI: 1.09–1.51) after adjusting for age, sex, and other vascular diseases related confounders. Our data also indicated that hyperhomocysteinemia could increase the risk of developing advanced carotid artery plaques by 28% in the population aged above 40.

In healthy population, the physiological range of plasma total homocysteine concentration is 5–15 µmol/L, and hyperhomocysteinemia (≥15 µmol/L) is considered to be pathological [18]. Previous cohort studies such as the Northern Manhattan cohort study, the Framingham Study and the British Regional Heart Study all showed that elevated homocysteine increases the risk of stroke [8], [11], [19]. Our results also suggest that carotid plaque development is associated with level of homocysteine, which may be indicative of the increased risk of stroke. In other words, formation of plaques in carotid artery walls can be an important risk factor for ischemic strokes [20]. It is widely accepted that about 60% vascular events are caused by rupture of vulnerable plaques that contain a large, thrombogenic core of lipid and necrotic debris [20].

Homocysteine is an amio acid in the plasma and plays an active role in oxide metabolism. It can also cause atherogenesis by impairing the inner vascular wall through different mechanisms, including damaging endothelial cells, enhancing platelet aggregation, reducing nitric oxide availability, promoting vascular smooth muscle cell proliferation, disturbing collagen synthesis, inducing chronic inflammation, or disrupting cholesterol and triglyceride biosynthesis [21]–[28]. Consequently, impairment of vascular wall may lead to breakdown of the integrity of the normal metabolism cycle, which could possibly change the plasma homocysteine concentration. Our results suggest that hyperhomocysteinemia can represent a sensitive predictor for carotid plaque development, particularly for the development of the advanced, unstable plaques in males. Future laboratory research is warranted to identify the biological involvement of homocysteine in plaque development and vascular injuries.

Nearly 50% participants in this study with carotid plaques had higher plasma homocysteine levels. Among those subjects with asymptomatic carotid plaques, their plasma homocysteine levels with vulnerable carotid plaques were much higher than in those with stable carotid plaques. Similarly, the NOMAS showed that elevated levels of homocysteine are independently associated with both echolucent, low-density plaques with low content of calcification and echodense and high-density plaques with high content of calcification [29]. Previous studies have shown that age and sex are the major determinants of concentration of plasma total homocysteine [30], [31]. Our study reported similar findings. We also found that the females had lower level of homocysteine as comparing to males. This could be due to the higher transmethylation rate in women [32]. Higher cigarettes consumption in men might be another contributing factor for the difference between male and female participants, which is consistent the findings from a study in Japan [30]. However, we could not exclude the fact that fewer female participants were enrolled than male participants in this study, and consequently the statistic power of the result found in females was weaker than in males. Thus, the analyses were further stratified by gender and age, respectively. Hyperhomocysteinemia in the male subjects were strongly related to risk of advanced carotid artery plaques, a phenomena that was not observed in the female subjects. In regard to risk of age, both middle-aged and elderly subjects with hyperhomocysteinemia demonstrated susceptibility to development of carotid plaque, and the ORs in subjects aged between 40–59 year old was slightly higher than the elderly subjects. Mechanisms need to be further explored in future studies.

Higher levels of homocysteine may be an independent risk factor of unstable carotid plaques. It is therefore speculated that strategies for reducing the plasma homocysteine levels in the middle-aged male population might be useful for stroke prevention. Several large clinical trials, however, did not support the use of folic acid and vitamin B supplements for prevention of recurrent stroke and transient ischemic attacks [33], [34]. Since homocysteine is influenced by nutritional status and genetic factors, difference in homocysteine levels between subjects in our study and other reports with Caucasians may be related to the different dietary habits or genetic backgrounds. Of note, the mean age of the studied populations in VISP [Vitamin Intervention for Stroke Prevention] and VITATOPS [VITAmins TO Prevent Stroke] trials were both over 65 years old [33], [34]. Another study suggests that in 66–69 years old population, homocysteine may not have a significant association with stroke occurrence [12].

In addition to the discoveries we have made in this study, the work had a few limitations. This is a cross-sectional study, which limits our ability to interpret the elevation of homocysteine level as a consequence or a causative factor of the development of carotid plaque. Also, we did not measure the plasma concentrations of serum folate and vitamin B_12_, which might affect the homocysteine level and potentially cause the bias. Further cohort studies are needed to further investigate these questions.

## Conclusion

In conclusion, the findings in this study show that higher level of total plasma homocysteine may be an independent risk factor for carotid plaque development in male Chinese adults. More epidemiological and experimental investigations are needed to explore whether increased homocysteine level plays a causative role in the advancement of advanced carotid plaque.
